# Effects of Water-Based Exercise on Patients Older than 60 Years Undergoing Cardiac Rehabilitation after Coronary Intervention

**DOI:** 10.3390/jcdd11050151

**Published:** 2024-05-15

**Authors:** Jus Ksela, Jan Kafol, Danijela Vasic, Borut Jug

**Affiliations:** 1 Department of Cardiovascular Surgery, University Medical Centre Ljubljana, 1000 Ljubljana, Slovenia; 2 Faculty of Medicine, University of Ljubljana, 1000 Ljubljana, Slovenia; jan.kafol@gmail.com (J.K.); borut.jug@kclj.si (B.J.); 3 Medical Centre Medicor, 6310 Izola, Slovenia; dottdanijela@gmail.com; 4 Department of Vascular Diseases, University Medical Centre Ljubljana, 1000 Ljubljana, Slovenia

**Keywords:** cardiac rehabilitation, coronary artery disease, exercise training, water-based (aquatic) exercise, VO_2_, heart rate variability

## Abstract

Cardiac rehabilitation (CR) plays a crucial role in managing patients who have undergone coronary intervention (CI) following acute myocardial infarction. While water-based exercise is gaining recognition as an exercise modality in this patient population, its impact on the subgroup of older adults remains unexplored. In this post hoc analysis, we investigated the effects of water-based exercise on adults older than 60 years undergoing CR after CI, comparing it to land-based exercise and a control group. In total, 45 patients aged over 60 participated in 14-day exercise programs, featuring two daily 30-min sessions. We assessed exercise capacity (VO_2peak_), vascular function (flow-mediated vasodilation (FMD)), heart rate variability (HRV), and blood markers (Interleukins 6, 8, and 10, P-Selectin, ICAM, and High-sensitivity CRP) before and after CR. VO_2peak_ in the water-based group improved significantly after CR in comparison with the land-based group: 1.35 kg/mL/min (95% CI [0.20–2.50], *p* = 0.022). The significant difference between water-based and land-based groups was observed in several HRV parameters: Total power −1129.20 ms^2^ (95% CI [−1951.92–−306.49], *p* = 0.008); peak LF 0.04 Hz (95% CI [0.00–0.08], *p* = 0.036); SD1 −9.02 millisecond (95% CI [−16.86–−1.18], *p* = 0.025); and SD2 −19.71 ms (95% CI [−35.08–−4.34], *p* = 0.013). FMD and blood markers did not vary significantly based on the exercise group. These findings suggest that short-term water-based CR may have potential as an alternative to traditional land-based CR, improving VO_2peak_ and cardiorespiratory fitness among adults over 60 years undergoing CR after CI.

## 1. Introduction

Coronary artery disease (CAD) stands as the predominant global cause of mortality, with age serving as an independent risk factor for cardiovascular disease [1]. Consequently, as the population ages, the prevalence of cardiovascular diseases rises, making the demand for accessible and efficient health services to tackle CAD paramount [2]. Among these services, exercise-based cardiac rehabilitation (CR) emerges as a cornerstone in comprehensive CAD management [3,4]. Cardiac rehabilitation, initially an exercise-focused program, has evolved into a comprehensive secondary prevention approach, addressing physical activity, risk reduction, education and promotion of healthy lifestyle in post-cardiovascular events [5]. Meta-analyses have demonstrated that exercise-based CR effectively reduces the risk of cardiovascular mortality, hospitalization, and myocardial infarction. Additionally, it enhances health-related quality of life and proves to be cost-effective [3,6,7]. Land-based aerobic and strength exercise training have traditionally dominated CR programs. However, alternative modalities, such as water-based exercises, are gaining traction [8].

Due to the limited understanding of the effects of water-based exercise in CR among older adults, we aimed to address this gap through appraising the effects of land- and water-based training modalities on exercise capacity, vascular function, cardiac autonomic modulation, preselected blood markers of inflammation, and endothelial dysfunction in CAD patients aged 60 and above who underwent CR within three months after coronary intervention (CI) following acute myocardial infarction.

## 2. Materials and Methods

This sub-analysis utilizes some of our collected data from a prospective, randomized, open-label parallel trial comparing water- and land-based exercise training with non-supervised training in CAD patients undergoing stationary cardiac rehabilitation, published previously by our research group [9,10].

### 2.1. Participants

The eligibility criteria for this study have been previously described [9,10]. CAD patients within three months post-myocardial infarction and revascularization (percutaneous coronary intervention (PCI) or coronary artery bypass graft (CABG)) undergoing stationary cardiac rehab were enrolled. Exclusions comprised uncontrolled/decompensated valve diseases, uncontrolled arterial hypertension, uncontrolled/high-risk dysrhythmias, a permanent pacemaker, exercise contraindications, inability to perform exercise testing, mental impairment, severe anemia, obstructive/restrictive lung disease, recent thromboembolic events, hepatic dysfunction, and an age of over 80 years. Eligible patients were invited to join this study. This study included 89 patients: 29 in the water-based group, 30 in the land-based group, and 30 in the control group [9,10].

For post hoc analysis, we selected the patients who were older than 60 years at the time that our study was conducted.

### 2.2. Intervention

The intervention was structured as either a water-based or land-based endurance plus calisthenics exercise program conducted over a 2-week residential CR. Each exercise program comprised 30-min training sessions held twice daily, six days a week, totalling 24 sessions. Before training, all participants underwent standard symptom-limited cardiopulmonary bicycle testing using the Schiller CS-200 cycle ergometer (Schiller A.G. Baar, Switzerland), with an incremental ramp protocol aiming for the predicted maximal workload. The test was considered complete if the respiratory exchange ratio was ≥1.1, and it was monitored continuously by ECG [9,10].

The water-based exercise regimen involved two daily sessions held in a heated swimming pool at 32.8 °C, with water depth adjusted to the xiphoid process level (1.5 m). Each session, lasting 30 min, comprised aerobic endurance and calisthenics. Aerobic endurance exercises began with a 5-min warm-up, followed by 20 min of conditioning activities like water walking, side-stepping, and arm cycling, performed at intensities ranging from 60% to 80% of the peak heart rate attained during symptom-limited graded exercise testing. The session concluded with a 5-min cool-down period. Calisthenics routines consisted of a 5-min warm-up, followed by 20 min of exercises targeting various upper and lower limb muscle groups, such as triceps extensions, triceps dips, modified leg press, leg abduction/adduction, and wall push-ups, all executed at 60–80% of the peak heart rate. Each calisthenics session also ended with a 5-min cool-down [10].

Land-based exercise training consisted of two 30-min sessions daily, incorporating bicycle ergometer training and calisthenics. The bicycle ergometer training session commenced with a 5-min warm-up, followed by 20 min at 60–80% of peak heart rate, and concluded with a 5-min cool-down. The calisthenics session began with a 5-min warm-up, followed by 20 min of exercises engaging muscle groups of the upper and lower limbs at 60–80% of peak heart rate, with a gradual increase in speed and repetitions, and concluded with a cool-down [10].

Patients in the control group were provided with lifestyle advice and educated about the advantageous effects of exercise. They were encouraged to partake in regular physical activities, such as walking, as part of their daily routines. However, they were instructed to abstain from enrolling in a supervised exercise program for the duration of the 2-week intervention period [10].

Other facets of rehabilitation, such as lifestyle education, implementation of a Mediterranean-style diet, medical oversight, and psychological assistance, were consistent for both intervention groups. Patients were accommodated in a specialized cardiac rehabilitation facility that offered comprehensive care, including prepared meals. Treatment protocols remained constant throughout the intervention period. However, adjustments to medication were allowed at the discretion of the attending cardiologist to maintain optimal control of risk factors [10].

### 2.3. Exercise Capacity

Aerobic exercise capacity was assessed by determining the VO2 peak using cardiopulmonary bicycle exercise testing conducted on a cycle-ergometer Schiller CS-200 (Schiller A.G. Baar, Switzerland) equipped with the Ganshorn Power Cube gas analysis unit (Ganshorn Deutschland GmbH, Niederlauer, Germany) [10].

Before each test, sensors were calibrated, and gas measurements (O_2_ and CO_2_) were taken. Participants underwent a symptom-limited exercise test, maintaining their usual routines and refraining from physical activities and heavy meals on the testing day. Resting-state data and ECG were recorded before the test. A maximal incremental protocol was employed, gradually increasing the workload on the computer-controlled cycle ergometer in a ramp-like fashion to reach the predicted maximal workload based on age, gender, and body surface area after 10 min. Test completion required a respiratory exchange ratio of ≥1.1. Participants wore a mouthpiece to measure oxygen and carbon dioxide flow (VO_2_ and VCO_2_). ECG, heart rate, and blood pressure were monitored at rest, at 2-min intervals during testing, during the cool-down period, and for 6 min after testing had concluded. No adverse effects were observed. To assess reproducibility, ten subjects were tested twice, yielding an intra-class correlation coefficient for a VO_2peak_ of 0.861 (*p* = 0.004) [10].

### 2.4. Vascular Function

Vascular function was assessed through an ultrasound examination of flow-mediated dilatation (FMD) of the right brachial artery, following standardized protocols and current guidelines [11]. This was conducted using a Philips ultrasound system iE 33, equipped with a high-resolution linear-array 10 MHz vascular probe [10].

The brachial artery was visualized at a location 2–10 cm above the elbow fossa. To assess endothelium-dependent vasodilation, a sphygmomanometer cuff on the forearm was inflated to 50 mmHg above systolic pressure. After releasing the pressure 4.5 min later, flow was measured within 15–20 s, and artery diameter within 60–90 s. Endothelium-independent dilation was induced by sublingual application of 0.4 mg nitroglycerin spray (Nitrolingual spray^®^) after a 15-min rest. Artery diameter and average blood flow velocity were then determined 3–4 min after dosing [10].

FMD was quantified as the percentage change from rest using the formula [(brachial artery diameter at peak hyperemia − diameter at rest) × 100/diameter at rest] [10].

### 2.5. Cardiac Autonomic Modulation

Before and after the intervention, patients underwent a 20-min high-resolution ECG recording using a 12-channel digital recorder (Schiller CS-200). Patients refrained from smoking or consuming caffeinated beverages 24 h before the recordings. Measurements were conducted between 9 and 11 AM in the fed state, following a short supine rest (10 min) in a controlled environment (quiet room, temperature: 22–24 °C, and humidity: 40–70%) [9].

The RR interval analysis began with offline processing using R-peak detection via the Schiller SEMA-200 system. Each detected beat was categorized as normal, ventricular ectopic, supraventricular ectopic, or unknown. R-wave peak locations were determined with a resolution of 1 ms through interpolation. Subsequently, an experienced observer manually reviewed and corrected all traces following automated editing. Abnormal beats, including those following ectopic beats in cases of ventricular or supraventricular ectopy, were eliminated without interpolation attempted for the removed intervals. A moving-window average filter was then applied to the edited data, computing a local average for each set of five contiguous NN intervals while excluding the central interval. Any central interval with a value 20% greater or smaller than the local average was considered an outlier and replaced by the local average. Only recordings with over 95% pure sinus beats were retained for linear and non-linear HRV analysis [9].

HRV analysis was conducted by processing raw data in Matlab (Math Work Inc., Natick, MA, USA), utilizing the open-source HRV Analysis Software (HRVAS) (available at http://sourceforge.net/projects/hrvas/, accessed on 5 July 2022), following methods outlined in the previous literature [12].

For non-linear HRV analysis we determined the standard deviation of the short- (SD1) and long-term (SD2) beat-to-beat RR interval variability measure (Poincaré plot), sample entropy (sampEn), and the short-term scaling exponent (Detrended fluctuation analysis, describing short-term fluctuations (DFA α1)) obtained by the Detrended fluctuation analysis technique of the RR interval time series (<11 beats) [9].

### 2.6. Blood Markers

Venous blood samples were analyzed for markers of low-grade inflammation (High-sensitivity C-reactive protein (hsCRP), Interleukin 6 (IL-6), Interleukin 8 (IL-8), Interleukin 10 (IL-10)) and endothelial activation (Intercellular adhesion molecule (ICAM), P-Selectin) [10].

Each participant provided fasting blood samples in the morning following a 30-min rest in the supine position. These samples were drawn from the cubital vein into 4.5 mL vacuum tubes containing 0.11 mol/L sodium citrate (Becton Dickinson, Vacutainer System Europe, Heidelberg, Germany). Plasma was prepared within 30 min through centrifugation at 2000× *g* for 20 min. It was later divided into aliquots, rapidly frozen in liquid nitrogen, and stored at –75 °C until further analysis. Plasma levels of hsCRP, ICAM-1, IL-6, IL-8, IL-10, and P-Selectin were assessed using xMAP^®^ Technology, which utilizes magnetic beads conjugated with specific antibodies (all from R&D Systems, Minneapolis, MN, USA), on a MagPix instrument (Luminex Corporation, Austin, TX, USA) [10].

### 2.7. Statistical Analysis

Data are presented as mean (±standard deviation) for normally distributed continuous variables, as median (interquartile range) if they lacked normal distribution, and as frequency (%) for categorical data. Between-group differences were evaluated using Fisher’s exact test, the Kruskal–Wallis rank sum test, or Pearson’s Chi-squared test, as appropriate. Differences between groups in the change of VO_2peak_, FMD, HRV parameters, and inflammatory markers were assessed using linear mixed-effects models, accounting for repeated measurements in each patient. Patients were included as random effects, with time (before/after intervention), group (land-based group, water-based group, and control), and time*group interaction designated as fixed effects. P-values were adjusted using false discovery rate correction for multiple testing and Bonferroni correction. Estimates were presented with 95% confidence intervals. A 2-tailed *p*-value < 0.05 was considered statistically significant. Statistical analyses were performed using R version 4.2.1 from the R Foundation for Statistical Computing.

## 3. Results

### 3.1. Baseline Characteristics

We included patients older than 60 years, which amounted to 45 patients from 89 included in the original study. In the land-based group, we had 20 participants, in the water-based group we had 11, and we had 14 in the control group. There were no significant differences in baseline characteristics between the three groups, except for prevalence of dyslipidemia, which was statistically significant between the control and land-based groups at the baseline (Table 1). However, almost all patients in all three groups were receiving lipid-lowering therapy as secondary prevention and were therefore comparable in terms dyslipidemia management [4].

### 3.2. Pre- and Post-Intervention Results

#### 3.2.1. Exercise Capacity and Vascular Function

There was a statistically significant difference (adj. *p* = 0.021) between the three groups in VO_2peak_ before the intervention. This was later accounted for in the mixed effects linear models for VO_2peak_. There were no statistically significant differences between the three groups in FMD pre- and post-intervention (Table 2).

**Figure 1:**
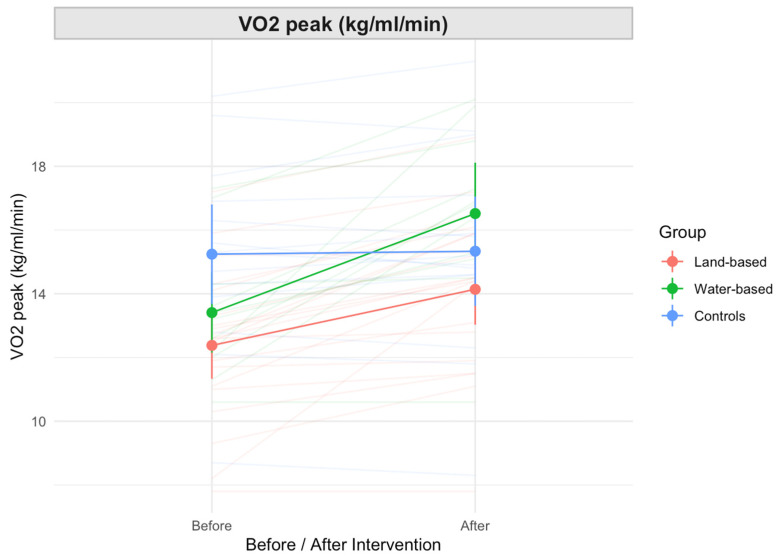
VO_2peak_ change before and after the intervention based on the exercise group.

VO_2peak_ changed significantly based on pre- and post-intervention and the exercise group (Figure 1). FMD did change significantly pre- and post-intervention, but did not vary significantly based on the exercise group (Table 3).

#### 3.2.2. Non-Linear HRV Parameters

There were no statistically significant differences between the three groups in non-linear HRV parameters pre- and post-intervention (Table 4).

There was a significant change in SD1 and SD2 between the land-based group and the water-based group after the intervention (Table 5).

#### 3.2.3. Blood Markers

There were no statistically significant differences between the three groups in blood markers pre- and post-intervention (Table 6).

There was a significant change in IL-6 and IL-8 in the land-based group before and after the intervention. There were no significant changes in blood markers between the groups (Table 7).

## 4. Discussion

Our study has shown that water-based exercise training is safe and effective for improving of exercise capacity (VO_2peak_) in patients with CAD aged 60 years or more. Although baseline VO_2peak_ was significantly different between the control and the land-based group, after taking into account individual variation of baseline capacity using mixed linear models, we confirmed a significant increase in VO_2peak_ induced by both training programs, particularly in water-based exercise training. These findings highlight the potentially beneficial effect of water-based CR in adults older than 60 years in enhancing cardiorespiratory fitness (CRF). Moreover, as an alternative to conventional land-based CR, water-based CR may even be more efficient in achieving CRF.

In older individuals, cardiovascular issues often coincide with age-related clinical complexities, such as multimorbidity, frailty, polypharmacy, cognitive dysfunction, and functional decline [13]. Managing cardiovascular disease in this demographic necessitates a paradigm shift towards individualized, patient-centered care, departing from strict adherence to guidelines. This approach prioritizes communication, risk–benefit balance, shared decision making, and addressing the patient’s health-related priorities, such as preserving function and quality of life, often despite limited support from the literature [14,15].

CR has demonstrated effectiveness for older adults with cardiovascular disease (CVD) and shows potential in addressing geriatric-specific complexities. However, functional, or cognitive challenges present barriers for many. Additionally, CR utilization declines with age due to referral and enrollment obstacles. Reengineering CR to address age-specific issues and embracing innovations like remote CR hold promise for improving effectiveness and access for older adults [16,17,18].

The psychological and mortality benefits observed after CR and exercise training programs are primarily linked to improvements in CRF [19]. VO_2peak_ measured via cardiopulmonary exercise testing remains the gold standard for assessing CRF [20]. Both VO_2peak_ and changes in VO_2peak_ have shown strong predictability for future risk of readmissions due to cardiovascular disease and all-cause mortality. Endpoint peak VO2 emerges as a robust and independent predictor of long-term survival in patients with CAD [21,22,23]. Following CR, elderly patients demonstrate larger relative enhancements in aerobic capacity compared to younger patients [18].

Water-based exercise was recognized as a safe and efficient intervention for cardiac patients. It has shown promising results in improving VO_2peak_, exercise time, and muscle strength in individuals with coronary artery disease. This suggests that water-based exercise could serve as a valuable strategy to enhance exercise capacity in patients with CAD and merits consideration for inclusion in CR programs [8,24]. Water-based exercise also offers unique advantages, including a low-risk environment that supports body weight, reduces the risk of falling, and minimizes joint overload and musculoskeletal injuries. These benefits may be particularly advantageous for older patients. However, the literature on the safety and effectiveness of water-based exercise in older adults is limited [8,25,26]. To our knowledge, our analysis is the first to demonstrate the potential benefits of water-based exercise in adults older than 60 years with CAD.

The short-term intervention period (i.e., 14-day duration of the training program) reflects prevalent clinical practices of CR in central Europe [27]. On the one hand, such time-intensive CR programs allows patients to dedicate their focus primarily to the rehabilitation process [27], and have shown effectiveness in eliciting favorable changes in cardiovascular physiological endpoints [28,29,30]. Our finding of improved exercise capacity after short-term CR indeed falls within this paradigm of time-intensive exercise training programmes. On the other hand, it is essential to acknowledge that this timeframe may not fully impact all physiological variables measured and longer intervention periods should be explored to comprehensively assess the effects of water-based CR in the older population on a broader range of parameters.

HRV refers to the variation in the time between two heartbeats and serves as a key indicator of various cardiovascular health conditions. It offers valuable insights into the interplay between the sympathetic and parasympathetic nervous systems, making it a useful tool for assessing autonomic nervous system functions [31]. Reduced HRV typically indicates heightened sympathetic activity, impaired autonomic regulation, and inadequate cardiovascular adaptation, while higher HRV suggests better autonomic control and optimal energy reserves. As a non-invasive marker, HRV aids in assessing the risk of cardiovascular events and mortality in patients with coronary artery disease. Different physical training modalities have shown positive effects on autonomic function leading to increased HRV parameters at rest [32,33,34].

Since previous studies revealed that both linear time- and frequency-domain HRV analysis lacks the ability to depict distinct changes in autonomic modulation in CAD patients undergoing CI, only non-linear HRV parameters were observed [9,35,36]. In the last decade, evidence has accumulated that DFA α1 offers a more precise estimate of sympathovagal balance and has been employed to evaluate cardiovascular risk, prognosis, and mortality in several studies [37,38]. Values of DFA α1 around 1.5 indicate self-similarity and inversely correlate with cardiovascular risk [39,40]. Our results showed that although DFA α1 did not significantly improve after CR, it demonstrated improvement in the water-based group and a tendency towards self-similarity. Although the described HRV changes in our analysis do not clearly demonstrate a superior effect of water-based exercise compared to conventional land-based CR, it is necessary to consider certain limitations of this study, as well as HRV as a prognostic marker, when interpreting them within the margin of our current study. The relatively brief intervention period of 14 days may have been insufficient to observe significant improvements in HRV parameters [32]. Hottenrott et al. suggested a minimum of 3 months of aerobic training to provoke alterations in vagal activity [41]. This was further reinforced by findings in a study conducted by Buchheit et al., which revealed no discernible changes in HRV during the initial 4-week period, indicating that this duration is inadequate to elicit improvements in autonomic function [42]. In light of these data, we could speculate that perhaps with a longer intervention period, a significant improvement of sympathovagal balance and consequently the complex HRV dynamics would have been observed, which would be line with the vast evidence of training-induced adaptations of cardiac autonomic function [9]. However, further studies are needed to present a more complete appraisal of the importance of duration of different exercise modalities on overall autonomic function restoration.

It is worth mentioning that recent research has shown that CR can also reduce levels of selected inflammatory and endothelial dysfunction markers [43,44]. However, in our analysis, the alterations in selected blood markers did not yield any additional insight into the favorability of water-based exercise compared to land-based exercise, as no significant differences were observed between the two groups after the intervention. Whether the 14-day exercise regime was indeed too short to show any significant effect on preselected biomarkers remains elusive and calls for further research.

Our study inherits several limitations from the original study, where these constraints were also outlined [9,10]. The post hoc analysis conducted in our study presents several additional limitations. Firstly, the retrospective nature of the analysis introduces inherent biases and constraints in establishing causality. Additionally, the reliance on existing data collected for another purpose may not fully capture all relevant variables or account for potential confounding factors. The relatively small sample size and uneven distribution of patients between the three groups may limit the generalizability of the findings. Moreover, individual responses to exercise varied (as depicted in Figure 1 by different slopes in the spaghetti plot graphs) and larger studies with more participants accounting for individual variation in response to exercise training are warranted. Finally, while post hoc analyses can generate hypotheses for future research, they cannot replace the rigor of prospective studies designed specifically to address the research question at hand. These limitations underscore the need for further investigation into the effects of water-based exercise in cardiac rehabilitation for older adults with CAD.

## 5. Conclusions

Our post hoc analysis highlights the potential advantages of short-term water-based CR in enhancing VO_2peak_ and cardiorespiratory fitness among patients older than 60 years with CAD. Our findings may contribute to uncovering the potential of water-based CR as a viable alternative to traditional land-based rehabilitation programs. However, further assessment of water-based CR in studies with larger samples is warranted to further evaluate it as an appropriate substitute for land-based CR.

## Figures and Tables

**Table 1:** Baseline characteristics.

Characteristic	Overall ^a^ (*n* = 45)	LB ^a^ (*n* = 20)	WB ^a^ (*n* = 11)	C ^a^ (*n* = 14)	*p* ^b^
Sex (male)	32 (71%)	12 (60%)	9 (82%)	11 (79%)	0.41
Age (years)	66.0 (63.0–70.0)	66.0 (62.0–70.5)	65.0 (62.5–68.0)	66.5 (64.0–71.5)	0.51
Smoking	23 (51%)	8 (40%)	5 (45%)	10 (71%)	0.18
Arterial hypertension	28 (62%)	12 (60%)	7 (64%)	9 (64%)	>0.99
Dyslipidemia	33 (73%)	11 (55%)	9 (82%)	13 (93%)	0.043 ^c^
Diabetes mellitus	6 (13%)	1 (5.0%)	2 (18%)	3 (21%)	0.29
Family history	29 (64%)	12 (60%)	8 (73%)	9 (64%)	0.92
Alcohol consumption	9 (20%)	4 (20%)	1 (9.1%)	4 (29%)	0.50
Physical inactivity	23 (51%)	11 (55%)	8 (73%)	4 (29%)	0.081
Obesity	9 (20%)	4 (20%)	3 (27%)	2 (14%)	0.80
BMI (kg/m^2^)	28.7 (26.8–30.7)	28.7 (26.5–30.1)	31.5 (28.9–33.5)	27.4 (26.5–29.6)	0.078
Antiplatelet	45 (100%)	20 (100%)	11 (100%)	14 (100%)	
Beta-blocker	40 (89%)	18 (90%)	10 (91%)	12 (86%)	>0.99
RAAS inhibitor	37 (82%)	15 (75%)	10 (91%)	12 (86%)	0.61
Lipid-lowering therapy	43 (96%)	19 (95%)	11 (100%)	13 (93%)	>0.99

^a^—*n* (%); median (IQR); ^b^—Fisher’s exact test; Kruskal–Wallis rank sum test; Pearson’s Chi-squared test; ^c^—LB vs. C; LB—land-based group; WB—water-based group; and C—control.

**Table 2:** VO_2peak_ and FMD pre- and post-intervention.

	Before				After			
Characteristic	LB (SD)	WB (SD)	C (SD)	adj. *p* ^a^	LB (SD)	WB (SD)	C (SD)	adj. *p* ^a^
VO_2peak_ (kg/mL/min)	12.38 (2.39)	13.41 (2.13)	15.24 (2.98)	0.021 ^b^	14.14 (2.53)	16.52 (2.70)	15.34 (3.28)	0.10
FMD (%)	6.4 (3.8)	8.0 (3.4)	7.0 (3.7)	0.55	10.9 (5.2)	9.6 (3.6)	9.0 (4.8)	0.55

^a^—adjusted *p*-value (*p* between the groups) with false discovery rate correction for multiple testing; ^b^—Bonferroni adjusted *p* < 0.05 for LB vs. C; LB—land-based group; WB—water-based group; C—control; SD—standard deviation; and FMD—Flow-mediated dilation.

**Table 3:** Mixed effect linear model for VO_2peak_ and FMD.

	VO_2peak_		FMD	
Predictors	Estimates (CI)	*p*	Estimates (CI)	*p*
LB_0_	12.38 (11.19–13.57)	<0.001	6.40 (4.52–8.28)	<0.001
WB_0_ vs. LB_0_	1.03 (−0.97–3.03)	0.309	1.61 (−1.55–4.76)	0.314
C_0_ vs. LB_0_	2.86 (1.01–4.72)	0.003	0.60 (−2.33–3.52)	0.686
LB_1_ vs. LB_0_	1.76 (1.08–2.44)	<0.001	4.49 (1.84–7.13)	0.001
WB_1_ vs. LB_1_	1.35 (0.20–2.50)	0.022	−2.89 (−7.32–1.55)	0.199
C_1_ vs. LB_1_	−1.67 (−2.73–−0.60)	0.003	−2.45 (−6.57–1.66)	0.239

LB—land-based group; WB—water-based group; C—control; CI—95% confidence interval; _0_—before the intervention; and _1_—after the intervention.

**Table 4:** Non-linear HRV Parameters pre-and post-intervention.

	Before				After			
Characteristic	LB (SD)	WB (SD)	C (SD)	adj. *p* ^a^	LB (SD)	WB (SD)	C (SD)	adj. *p* ^a^
SD1 (ms)	16 (10)	18 (14)	18 (16)	0.98	18 (11)	11 (6)	19 (16)	0.22
SD2 (ms)	46 (17)	57 (24)	50 (22)	0.98	54 (15)	45 (15)	46 (18)	0.25
SampEn	2.03 (0.36)	1.93 (0.52)	2.13 (0.45)	0.98	1.91 (0.40)	1.89 (0.36)	2.16 (0.35)	0.22
DFA α1	1.10 (0.28)	1.29 (0.28)	1.05 (0.32)	0.61	1.15 (0.26)	1.34 (0.21)	1.01 (0.30)	0.12

^a^—adjusted *p*-value (*p* between the groups) with false discovery rate correction for multiple testing; LB—land-based group; WB—water-based group; C—control; SD—standard deviation; SD1—Poincaré plot standard deviation perpendicular to the line of identity; SD2—Poincaré plot standard deviation along the line of identity; SampEn—sample entropy; and DFA α1—Detrended fluctuation analysis, describing short-term fluctuations.

**Table 5:** Mixed effect linear model for non-linear HRV parameters.

	SD1		SD2	
Predictors	Estimates (CI)	*p*	Estimates (CI)	*p*
LB_0_	15.80 (10.31–21.29)	<0.001	46.19 (38.05–54.34)	<0.001
WB_0_ vs. LB_0_	2.18 (−7.03–11.40)	0.639	10.48 (−3.20–24.16)	0.131
C_0_ vs. LB_0_	2.23 (−6.32–10.78)	0.606	4.09 (−8.61–16.79)	0.523
LB_1_ vs. LB_0_	2.42 (−2.25–7.09)	0.306	8.23 (−0.93–17.39)	0.077
WB_1_ vs. LB_1_	−9.02 (−16.86–−1.18)	0.025	−19.71 (−35.08–−4.34)	0.013
C_1_ vs. LB_1_	−1.50 (−8.78–5.78)	0.683	−12.58 (−26.85–1.69)	0.083
	**SampEn**		**DFA α1**	
**Predictors**	**Estimates (CI)**	***p***	**Estimates (CI)**	***p***
LB_0_	2.03 (1.86–2.21)	<0.001	1.10 (0.98–1.23)	<0.001
WB_0_ vs. LB_0_	−0.10 (−0.41–0.20)	0.491	0.18 (−0.02–0.39)	0.084
C_0_ vs. LB_0_	0.09 (−0.19–0.37)	0.514	−0.06 (−0.25–0.14)	0.564
LB_1_ vs. LB_0_	−0.13 (−0.35–0.10)	0.268	0.05 (−0.06–0.16)	0.394
WB_1_ vs. LB_1_	0.08 (−0.29–0.46)	0.657	0.01 (−0.19–0.20)	0.958
C_1_ vs. LB_1_	0.16 (−0.19–0.51)	0.364	−0.09 (−0.27–0.09)	0.325

LB—land-based group; WB—water-based group; C—control; CI—95% confidence interval; _0_—before the intervention; _1_—after the intervention; SD1—Poincaré plot standard deviation perpendicular to the line of identity; SD2—Poincaré plot standard deviation along the line of identity; SampEn—sample entropy; and DFA α1—Detrended fluctuation analysis, describing short-term fluctuations.

**Table 6:** Inflammatory markers pre-and post-intervention.

	Before				After			
Characteristic	LB (SD)	WB (SD)	C (SD)	adj. *p* ^a^	LB (SD)	WB (SD)	C (SD)	adj. *p* ^a^
IL-6 (ng/L)	9.19 (8.50)	7.54 (1.75)	9.08 (2.69)	0.79	8.53 (7.35)	7.06 (1.71)	8.33 (1.99)	0.52
IL-8 (ng/L)	19.4 (4.2)	18.1 (3.6)	19.0 (4.1)	0.79	18.09 (3.71)	17.46 (3.41)	18.01 (3.49)	0.86
IL-10 (ng/L)	13.4 (3.0)	13.8 (3.2)	15.7 (5.1)	0.79	12.7 (3.1)	12.9 (2.7)	14.9 (3.7)	0.52
P-Selectin (micg/L)	26.7 (6.2)	25.1 (5.1)	27.2 (5.9)	0.79	25.2 (6.0)	24.6 (5.6)	26.7 (5.7)	0.86
ICAM (micg/L)	794 (401)	576 (216)	740 (444)	0.79	785 (393)	577 (222)	749 (443)	0.86
hsCRP (mg/L)	1.22 (1.47)	1.07 (0.76)	1.34 (1.46)	0.90	1.26 (1.67)	1.11 (1.12)	1.21 (1.32)	0.96

^a^—adjusted *p*-value (*p* between the groups) with false discovery rate correction for multiple testing; LB—land-based group; WB—water-based group; C—control; SD—standard deviation; IL-6—Interleukin 6; IL-8—Interleukin 8; IL-10—Interleukin 10; ICAM—Intercellular adhesion molecule; and hsCRP—High-sensitivity C-reactive protein.

**Table 7:** Mixed effect linear model for inflammatory markers.

	IL-6		IL-8		IL-10	
Predictors	Estimates (CI)	*p*	Estimates (CI)	*p*	Estimates (CI)	*p*
LB_0_	9.18 (6.71–11.66)	<0.001	19.42 (17.73–21.11)	<0.001	13.35 (11.78–14.92)	<0.001
WB_0_ vs. LB_0_	−1.65 (−5.81–2.51)	0.433	−1.34 (−4.17–1.50)	0.350	0.45 (−2.18–3.08)	0.734
C_0_ vs. LB_0_	−0.11 (−3.97–3.75)	0.956	−0.39 (−3.02–2.24)	0.768	2.36 (−0.08–4.81)	0.057
LB_1_ vs. LB_0_	−0.66 (−1.28–−0.04)	0.038	−1.33 (−2.44–−0.23)	0.018	−0.67 (−1.67–0.33)	0.184
WB_1_ vs. LB_1_	0.19 (−0.86–1.23)	0.722	0.72 (−1.13–2.57)	0.443	−0.21 (−1.88–1.46)	0.802
C_1_ vs. LB_1_	−0.09 (−1.06–0.88)	0.854	0.32 (−1.40–2.04)	0.711	−0.18 (−1.73–1.37)	0.818
	**P-Selectin**		**ICAM**		**hsCRP**	
**Predictors**	**Estimates (CI)**	***p***	**Estimates (CI)**	***p***	**Estimates (CI)**	***p***
LB_0_	26.69 (24.10–29.28)	<0.001	794.15 (625.69–962.61)	<0.001	1.22 (0.60–1.84)	<0.001
WB_0_ vs. LB_0_	−1.55 (−5.91–2.80)	0.480	−217.70 (−500.50–65.11)	0.130	−0.15 (−1.19–0.89)	0.776
C_0_ vs. LB_0_	0.55 (−3.49–4.59)	0.789	−53.79 (−316.32–208.74)	0.685	0.12 (−0.85–1.08)	0.807
LB_1_ vs. LB_0_	−1.47 (−2.95–0.01)	0.051	−9.60 (−23.68–4.48)	0.179	0.05 (−0.46–0.55)	0.858
WB_1_ vs. LB_1_	0.94 (−1.54–3.42)	0.452	10.05 (−13.58–33.69)	0.400	−0.01 (−0.85–0.84)	0.985
C_1_ vs. LB_1_	0.96 (−1.35–3.26)	0.411	17.96 (−3.99–39.90)	0.107	−0.17 (−0.96–0.61)	0.663

LB—land-based group; WB–water-based group; C—control; CI—95% confidence interval; _0_—before the intervention; _1_—after the intervention; IL-6—Interleukin 6; IL-8—Interleukin 8; IL-10—Interleukin 10; ICAM—Intercellular adhesion molecule; and hsCRP—High-sensitivity C-reactive protein.

## Data Availability

The data used in the analysis are available on request to the corresponding author.
